# 

**Published:** 1886-10-20

**Authors:** 


### TABLE OF CONTENTS.

### Original and Selected Articles.

Bite of a Copperbelly Snake, by A. F. Durham, M.D., of Georgia....365
Paraplegia from Reflex Causes, by S. T. DeLa Mater, M D., Fayetteville, N. Y....367
Prophylactic Treatment of Abortion, by J. H. Coulter, A.M., M.D . Peoria, Ill....369
The Treatment of Gonorrhœa, by Seneca D. Powell. M.D....371
Tests for Impurities in Water Available for Physicians' Use, by Curtis C. Howard, M.C....374
Chlorate of Potassium in the Treatment of Hemorrhoids....375
Suppurating Veneral Bubo; with Remarks on a Method of Subcutaneous Treatment and Report of Cases, by Scott Helm, M.D....376
The Baby as a Water-Drinker, by C. A. Bryce, M D., Richmond, Va....379
Acute Rheumatism; Novel Treatment of Warts....380

### Abstracts and Gleanings.

Varicocele....381
Ophthalmology in the London Ophthalmic Hospital.... 302
Urethran Ethyl Carbonate as a Physilogical Antidote to Strychnine.... 385
Creasote a Specific for Erysipelas....386
Typhoid Fever....387
The Hypodermic Injection of Morphia in Controlling Convulsions in Children....388
Hot Bath, Hot Rock, and Pilocarpine Compared....389
On Lobelia Inflata in Asthma....390
Cocaine in Cata act Extraction; Conception of Male Children at the Time of the Post-Menstrual Anæmia....391
Cholera Infantum; On Revaccinaiion....392
Carbolic Treatment of Hemorrhoids; Substitute for Circumcision; Cocaine to Diminish Labor Pains....393
Delivery During Hypnosis; Jaborandi in Erysipelas; Post-Partum Hemorrhage....394

### Scientific Items.

Cause of the Charleston Earthquake; Influence of Magnetism on Chemical Reaction....395
Machine to Make Conductors Honest; Electrical Foot Warmers....397

### Practical Notes and Formulæ.

For Diarrhœa; Scarlet Fever and Diphtheria 399

### Editorials and Miscellaneous.

Subscribers in Arrears; A Liberal Offer; the Earthquake at Charleston....398
Proposition to Change the Organization of the Medical Association of Georgia....399
Southern Medical College—Opening Exercises..400
Books and Pamphlets Received....403
Receipts; Special Notices....404

